# Deep learning models for radiography body-part classification and chest radiograph projection/orientation classification: a multi-institutional study

**DOI:** 10.1007/s00330-025-12053-7

**Published:** 2025-10-22

**Authors:** Yasuhito Mitsuyama, Hirotaka Takita, Shannon L. Walston, Ko Watanabe, Shoya Ishimaru, Yukio Miki, Daiju Ueda

**Affiliations:** 1 Department of Diagnostic and Interventional Radiology, Graduate School of Medicine, Osaka Metropolitan University, 1-4-3 Asahi-Machi, Abeno-ku, Osaka, 545-8585 Japan; 2 Department of Artificial Intelligence, Graduate School of Medicine, Osaka Metropolitan University, 1-4-3 Asahi-Machi, Abeno-ku, Osaka, 545-8585 Japan; 3 Smart Data and Knowledge Services Department, German Research Center for Artificial Intelligence (DFKI GmbH), 67663 Kaiserslautern, Germany; 4 Department of Core Informatics, Graduate School of Informatics, Osaka Metropolitan University, 1-1, Gakuen-cho, Naka-ku, Sakai, 599-8531 Japan; 5 Center for Health Science Innovation, Osaka Metropolitan University, 1-4-3, Asahi-Machi, Abeno-ku, Osaka, 545-8585 Japan

**Keywords:** Deep learning, Radiography (thoracic), Image processing (computer-assisted), Quality control, Multicenter study

## Abstract

**Objectives:**

Large-scale radiographic datasets often include errors in labels such as body parts or projection, which can undermine automated image analysis. Therefore, we aimed to develop and externally validate two deep-learning models—one for categorising radiographs by body part, and another for identifying projection and rotation of chest radiographs—using large, diverse datasets.

**Materials and methods:**

We retrospectively collected radiographs from multiple institutions and public repositories. For the first model (Xp-Bodypart-Checker), we included seven categories (Head, Neck, Chest, Incomplete Chest, Abdomen, Pelvis, Extremities). For the second model (CXp-Projection-Rotation-Checker), we classified chest radiographs by projection (anterior-posterior, posterior-anterior, lateral) and rotation (upright, inverted, left rotation, right rotation). Both models were trained, tuned, and internally tested on separate data, then externally tested on radiographs from different institutions. Model performance was assessed using overall accuracy (micro, macro, and weighted) as well as one-vs.-all area under the receiver operating characteristic curve (AUC).

**Results:**

In the Xp-Bodypart-Checker development phase, we included 429,341 radiographs obtained from Institutions A, B, and MURA. In the CXp-Projection-Rotation-Checker development phase, we included 463,728 chest radiographs from CheXpert, PadChest, and Institution A. The Xp-Bodypart-Checker achieved AUC values of 1.00 (99% CI: 1.00–1.00) for all classes other than Incomplete Chest, which had an AUC value of 0.99 (99% CI: 0.98–1.00). The CXp-Projection-Rotation-Checker demonstrated AUC values of 1.00 (99% CI: 1.00–1.00) across all projection and rotation classifications.

**Conclusion:**

These models help automatically verify image labels in large radiographic databases, improving quality control across multiple institutions.

**Key Points:**

***Question*** *This study examines how deep learning can accurately classify radiograph body parts and detect chest projection/orientation in large, multi-institutional datasets, enhancing metadata consistency for clinical and research workflows.*

***Findings*** *Xp-Bodypart-Checker classified radiographs into seven categories with AUC values of over 0.99 for all classes, while CXp-Projection-Rotation-Checker achieved AUC values of 1.00 across all projection and rotation classifications*.

***Clinical relevance*** *Trained on over 860,000 multi-institutional radiographs, our two deep-learning models classify radiograph body-part and chest radiograph projection/rotation, identifying mislabeled data and enhancing data integrity, thereby improving reliability for both clinical use and deep-learning research*.

**Graphical Abstract:**

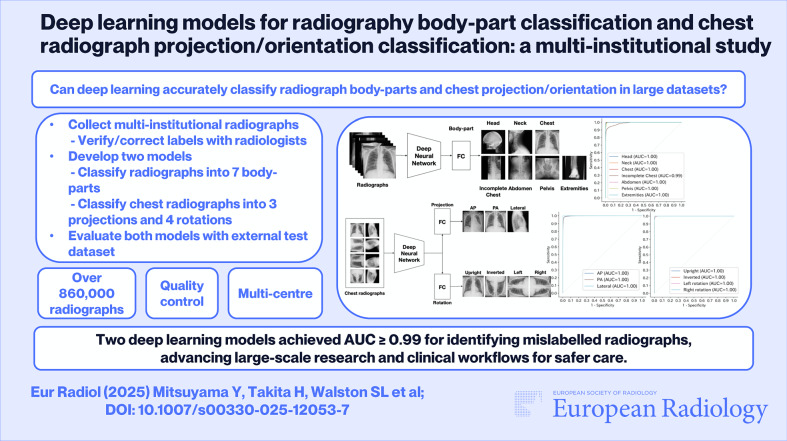

## Introduction

Deep-learning (DL) models using chest radiographs have made remarkable progress in recent years [1, 2]. We are now at a stage where more scalable and versatile models are being developed using larger, multi-institutional, and multinational datasets. Beyond disease detection, these models have evolved to accomplish tasks that are challenging for humans, such as estimating cardiac function, respiratory function, and biological age, offering substantial promise for improving the diagnosis of chest radiographs [3–5]. Consequently, there is a growing demand for more diverse datasets to power these advanced applications.

However, with the diversification and expansion of data, quality control for model training has emerged as a crucial challenge. As chest radiograph repositories increase in size and heterogeneity, ensuring correct labeling becomes more difficult, particularly because many images are stored in DICOM format, which relies on human entry for patient and examination information [6]. In practice, errors and missing data frequently arise from manual or semi-automated workflows [7, 8]. Basic labels indicating the body part and projection are critical both for clinical settings and data preprocessing [9]. Furthermore, it is often observed that radiograph databases contain images with various rotations. Such basic labeling inaccuracies and the inclusion of images with various rotations may compromise the reliability of deep-learning-based analyses. These issues highlight the need for automated solutions that can detect mislabeled images before they negatively affect training and validation in deep-learning pipelines.

To address these challenges, several machine learning and deep-learning methods have been proposed for classifying radiographed body parts, chest radiograph projection, or chest radiograph rotation [10–21]. These automated systems aim to enhance standardisation by catching potential metadata errors. However, many of these earlier studies relied on single-institution data or relatively small datasets, limiting their generalizability [11–19]. Broader validation across multiple healthcare systems and regions is therefore vital to establish these models’ effectiveness in diverse clinical settings.

Against this background, we developed and externally tested two separate deep-learning models using a large-scale, multi-institutional dataset. The first model, Xp-Bodypart-Checker, classifies radiographs of various body parts, while the second model, CXp-Projection-Rotation-Checker, specialises in detecting the projection and rotation of chest radiographs.

## Methods

### Study design

This multicentre retrospective study was conducted to develop and externally test two distinct DL models. The first model, Xp-Bodypart-Checker, classifies body parts from various body-part radiographic images. The second model, CXp-Projection-Rotation-Checker, determines both the projection (anterior-posterior (AP), posterior-anterior (PA), or lateral) and the rotation (upright, inverted, left rotation, or right rotation) of chest radiographs. The study followed the Declaration of Helsinki and was approved by the Institutional Review Board (approval numbers 2024-100 and 2022-0021 K). The requirement for informed consent was waived by the ethics committees because only routinely acquired clinical images were used. This manuscript was prepared in compliance with the STARD guidelines [22].

### Participants, image collection, and examination acquisition

For Xp-Bodypart-Checker, radiographs of patients aged ≥ 18 years were retrospectively collected at Institution A (January 1, 2019, to December 31, 2021) and Institution B (January 1, 2023, to December 31, 2023). All radiographic examinations for each patient in this period were included. Additionally, upper extremity radiographs were collected from MURA [23]. MURA is a large dataset of musculoskeletal radiographs containing 40,561 images from 14,863 studies. As age information was not available in MURA, all publicly available radiographs from this database were collected.

For CXp-Projection-Rotation-Checker, chest radiographs of patients aged ≥ 18 years were consecutively collected from Institution A (January 1, 2019, to December 31, 2021), as well as CheXpert [24] and PadChest [25]. CheXpert is one of the largest publicly available chest radiograph datasets, comprising 224,316 chest radiographs from 65,401 patients who underwent radiographic examinations at Stanford Health Care between October 2002 and July 2017. The PadChest dataset is composed of 160,861 images from over 67,000 patients, collected from Hospital San Juan in Spain from 2009 to 2017. Since PadChest did not directly record patient age at examination, we excluded patients under 18 years old by calculating age from the “StudyDate_DICOM” and “PatientBirth” columns, and also excluded any paediatric protocols, corrupted images, or unknown birth years. If a patient had multiple, inconsistent gender entries, we labeled gender as unknown. All radiographic examinations for each patient in this period were included.

### Reference standard

For Xp-Bodypart-Checker, initially, all radiographs from Institutions A and B were classified into six categories (Head, Neck, Chest, Abdomen, Pelvis, and Extremities) based on the BodyPartExamined tag in their DICOM metadata, then verified and corrected by two board-certified radiologists (5 and 9 years of experience). Spinal radiographs were categorised so that cervical spine images were assigned to “Neck”, thoracic spine images to “Chest”, and lumbar spine images to “Abdomen”. To further refine the classification for images labeled as “Chest,” we examined whether both lung fields were fully captured or partly excluded, thereby subdividing this category into “Chest,” in which both lung fields were fully visible, and “Incomplete Chest,” in which one or both lung fields were partially excluded. Additionally, all radiographs from MURA were labeled as “Extremities”. As a result, the final classification encompassed seven distinct categories in total.

For CXp-Projection-Rotation-Checker, chest radiographs from Institution A were classified into three projections (AP, PA, and Lateral) based on the BodyPartExamined tag, ProtocolName tag, SeriesDescription tag, and StudyDescription tag in their DICOM metadata, then verified and corrected by two board-certified radiologists (5 and 9 years of experience). Using the imaging data recorded in each database (CheXpert and PadChest), we classified the chest radiographs according to their projection. Furthermore, rotation labels were randomly assigned to all chest radiographs to achieve an equal distribution (1:1:1:1 ratio) among four rotations: upright, inverted, left rotation, and right rotation. The images were then rotated according to these assigned rotation labels. Neither Institution A nor Institution B contributed data to CheXpert, PadChest, or MURA.

### Data partition

For Xp-Bodypart-Checker, radiographs from Institution A were randomly allocated on a per-patient basis in an 8:1:1 ratio for training, tuning, and internal test datasets, respectively. All radiographs from Institution B and MURA were utilised as the external test dataset.

For CXp-Projection-Rotation-Checker, chest radiographs from CheXpert and PadChest were randomly allocated on a per-patient basis in an 8:1:1 ratio for training, tuning, and internal test datasets, respectively. All chest radiographs from Institution A were utilised as the external test dataset.

### Model development

**Fig. 1:**
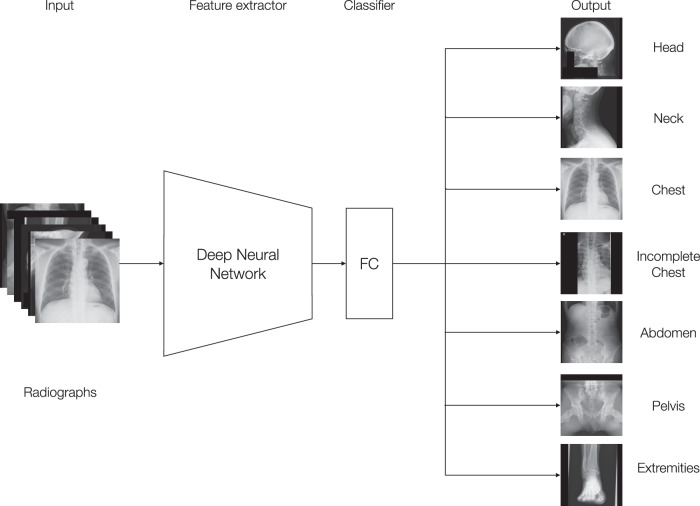
Outline of Xp-Bodypart-Checker. The input various body-part radiograph is fed into an EfficientNet-based feature extractor, which progressively down-samples the image through multiple convolutional layers to learn high-level features. The final extracted feature map is then passed into a fully connected (FC) layer that serves as the classifier. This FC layer outputs one of seven body-part categories for the input radiographs

**Fig. 2:**
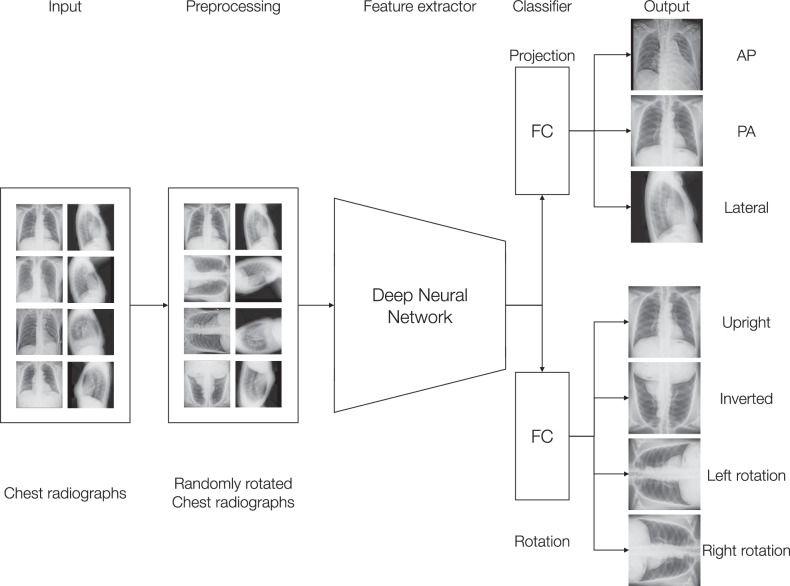
Outline of CXp-Projection-Rotation-Checker. The input chest radiograph is fed into an EfficientNet backbone, which progressively down-samples and extracts hierarchical image features. The resulting feature representation is then passed into a fully connected (FC) layer configured to produce two classification outputs: one for chest radiograph projection (e.g., anterior-posterior, posterior-anterior, or lateral) and another for chest radiograph rotation (e.g., upright, inverted, left rotation, or right rotation). This multi-output design allows the model to learn both tasks simultaneously from a shared set of features

We developed two DL models using EfficientNet [26]: one for classifying radiograph body parts (Xp-Bodypart-Checker) and another for classifying projection and rotation of chest radiographs (CXp-Projection-Rotation-Checker). Cross-entropy loss was used as the loss function for both models. Each DL model was trained and tuned using the training and tuning datasets, respectively, starting with the initial parameters from the ImageNet-pretrained model and allowing the overall parameters to be updated. The model that achieved the smallest loss function value on the tuning dataset (within 100 epochs) was selected as the best-performing model. The longest side of each image was downscaled to 256 pixels while maintaining the aspect ratio, and the width along the shorter side was then padded with black to 256 pixels. We performed data expansion with TrivialAugment Wide [27]. Every development process was performed using the PyTorch framework (version 2.0.1). To compare performance, we additionally implemented two baseline approaches: a convolutional neural network using ResNet‑50 [28] and a classical computer‑vision pipeline that combines Histogram of Oriented Gradients (HOG) feature extraction with a linear Support Vector Machine (SVM) classifier [29]. Detailed processes for the development of the models and machine environment are shown in the Supplementary appendix (p 2). Outlines of each of the two models are shown in Figs. 1 and 2.

### Model test

For Xp-Bodypart-Checker, we evaluated the classification performance of the DL model using the best model identified by the tuning dataset. The accuracy of body-part classification from radiographs was evaluated using an internal test dataset (Institution A) and external test datasets (Institution B and MURA). An external test was performed on the dataset from Institution B and MURA, in which images were zero‑padded by 48 pixels on all four sides to remove any side markers. Saliency maps were generated for each radiograph in the external test dataset from institution B to indicate the region of interest used in the classification of the body part. Shapley Additive exPlanations (SHAP), a framework that applies the Shapley value (an importance value calculated based on game theory) to machine learning, was used to generate saliency maps for each radiograph [30]. For a computer vision task, SHAP computes the contribution of a group of pixels to a particular prediction.

For CXp-Projection-Rotation-Checker, we evaluated the classification performance of the DL model using the best model identified by the tuning dataset. The accuracy of projection classification and rotation classification from chest radiographs was evaluated using internal test datasets (CheXpert and PadChest) and an external test dataset (Institution A). External testing was performed using real rotation labels from Institution A. External test images from Institution A were zero‑padded by 48 pixels on all four sides to remove any side markers. Saliency maps were generated for each radiograph in the external test dataset from institution A to indicate the region of interest used in the classification of the projection and rotation.

### Statistical analysis

For Xp-Bodypart-Checker, due to class imbalance in the dataset, overall model performance was evaluated using three accuracy metrics: micro-average accuracy, calculated as the ratio of the total number of correctly classified radiographs to the total number of radiographs across all classes; macro-average accuracy, defined as the unweighted mean of the accuracies for each class; and weighted-average accuracy, computed as the mean of class-specific accuracies weighted by the number of radiographs in each class. Furthermore, we calculated the accuracy for each body-part class and generated receiver operating characteristic (ROC) curves and area under the receiver operating characteristic curve (AUC) values. We created confusion matrices for the external test datasets (Institution B and MURA). Furthermore, we calculated the accuracy and AUC values using the Institution B dataset, which contained incorrect body-part labels.

For CXp-Projection-Rotation-Checker, due to class imbalance in the dataset, overall model performance was evaluated using three accuracy metrics for both projection and rotation classification tasks: micro-average accuracy, calculated as the ratio of the total number of correctly classified radiographs to the total number of radiographs across all classes; macro-average accuracy, defined as the unweighted mean of the accuracies for each class; and weighted-average accuracy, computed as the mean of class-specific accuracies weighted by the number of radiographs in each class. Additionally, we calculated the accuracy for each projection class and rotation class, and generated ROC curves and AUC values. We created confusion matrices for the external test dataset (Institution A) for both projection and rotation classification tasks. We calculated the accuracy and AUC values using the Institution A dataset, which contained incorrect projection labels.

All analyses were done in SciPy using Python (version 3.8.1). These statistical inferences were performed with a two-sided significance level of 1%, and the performance metrics were presented with 99% confidence intervals.

## Results

### Datasets

**Fig. 3:**
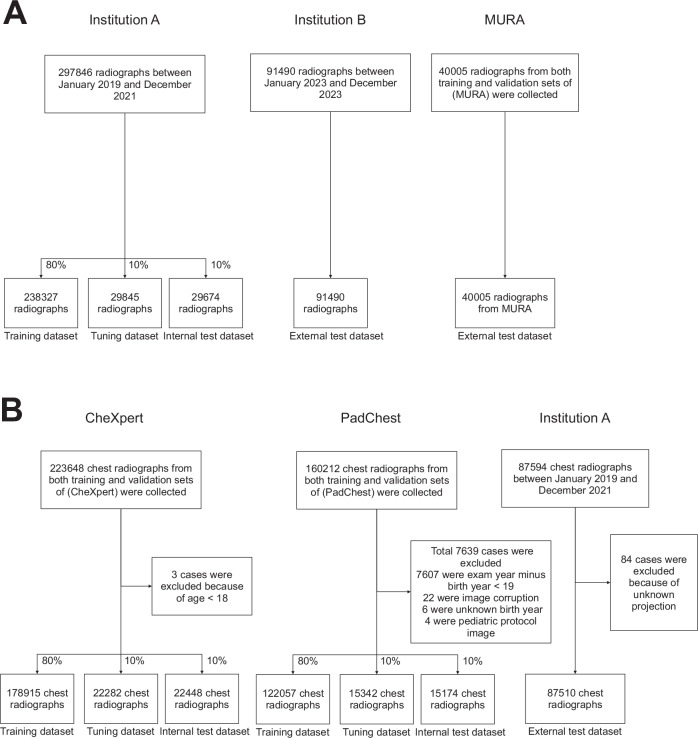
Eligibility flowcharts of Xp-Bodypart-Checker and CXp-Projection-Rotation-Checker. **A** These flowcharts describe the training, tuning, internal test, and external test datasets used during the development phase of Xp-Bodypart-Checker. **B** These flowcharts describe the training, tuning, internal test, and external test datasets used in the development phase of CXp-Projection-Rotation-Checker

For Xp-Bodypart-Checker, we included 297,846 radiographs obtained from 44,772 patients at Institution A, 91,490 radiographs from 12,895 patients at Institution B, and 40,005 radiographs from MURA. Details of these datasets are shown in Table 1, and the eligibility flowcharts are illustrated in Fig. 3A. After review by two radiologists, the body-part labels based on the DICOM tags were modified for 3.9% of the radiographs from Institution A and 12.8% of those from Institution B, as summarised in the Supplementary appendix (p 3).

**Table 1:** Demographics of datasets for Xp-Bodypart-Checker

	Institution A			Institution B	MURA
	Training	Tuning	Internal test	External test	External test
Total radiographs	237,475	31,039	29,332	91,490	40,005
Total patients	33,388	5715	5669	12,895	N/A
Male	15,376	2575	2508	5308	N/A
Female	18,012	3140	3161	7587	N/A
Age (years)					
Mean ( ± sd)	62 ± 17.2	63 ± 16.7	63 ± 16.7	71 ± 14.3	N/A
Range	18–105	18–101	18–98	18–102	N/A
Body part shown in radiograph					
Head	1997	268	235	587	0
Neck	15,623	1829	1952	2652	0
Chest	69,560	9221	8813	26,370	0
Incomplete chest	7797	1058	1058	655	0
Abdomen	68,018	8645	8164	12,468	0
Pelvis	17,417	2443	1927	11,613	0
Extremities	57,063	7575	7183	37,145	40,005

Data are no. unless otherwise noted. sd denotes standard deviation

For CXp-Projection-Rotation-Checker, we included 223,645 chest radiographs from 64,737 patients in CheXpert after excluding 3 chest radiographs for not meeting the age criterion. We included 152,573 chest radiographs from 62,944 patients in PadChest, with 7639 chest radiographs excluded. The majority of these (7613 chest radiographs) were excluded for not meeting the age criterion, including 7607 radiographs for which the examination year minus the patient’s birth year was < 19, and 6 radiographs for which the birth year was unknown. Another 22 chest radiographs were excluded due to image corruption, and 4 chest radiographs were excluded because they were acquired under a paediatric protocol. We included 87,510 chest radiographs from 25,228 patients at Institution A, after excluding 84 with an indeterminable projection. Details of these datasets are shown in Table 2, and the eligibility flowcharts are illustrated in Fig. 3B. After review by two radiologists, the projection labels based on the DICOM tags were modified for 3.3% of the chest radiographs from Institution A, as summarised in the Supplementary appendix (p 4).

**Table 2:** Demographics of datasets for CXp-Projection-Rotation-Checker

	CheXpert			PadChest			Institution A
	Training	Tuning	Internal test	Training	Tuning	Internal test	External test
Total	180,002	21,894	21,749	121,910	15,509	15,154	87,510
Total patients	51,980	6398	6359	50,337	6376	6231	25,228
Male	28,837	3564	3516	24146	3020	2964	12,693
Female	23,142	2834	2843	26,182	3356	3267	12,535
Unknown	1	0	0	9	0	0	0
Age (years)							
Mean ( ± sd)	60 ± 18.6	60 ± 18.5	60 ± 18.4	59 ± 17.5	59 ± 17.5	59 ± 17.6	63 ± 16.8
Range	18–90	18–90	18–90	19–105	19–100	19–104	18–101
Projection							
Frontal	154,077	18,739	18,393	83,743	10,657	10,406	78,184
AP	130,521	15,900	15,335	13,444	1903	1626	27,803
PA	23,556	2839	3058	70,299	8754	8780	50,381
Lateral	25,925	3155	3356	38,167	4852	4748	9326

Data are no. unless otherwise noted. sd denotes standard deviation

*AP* anterior-posterior, *PA* posterior-anterior

### Model evaluation

**Fig. 4:**
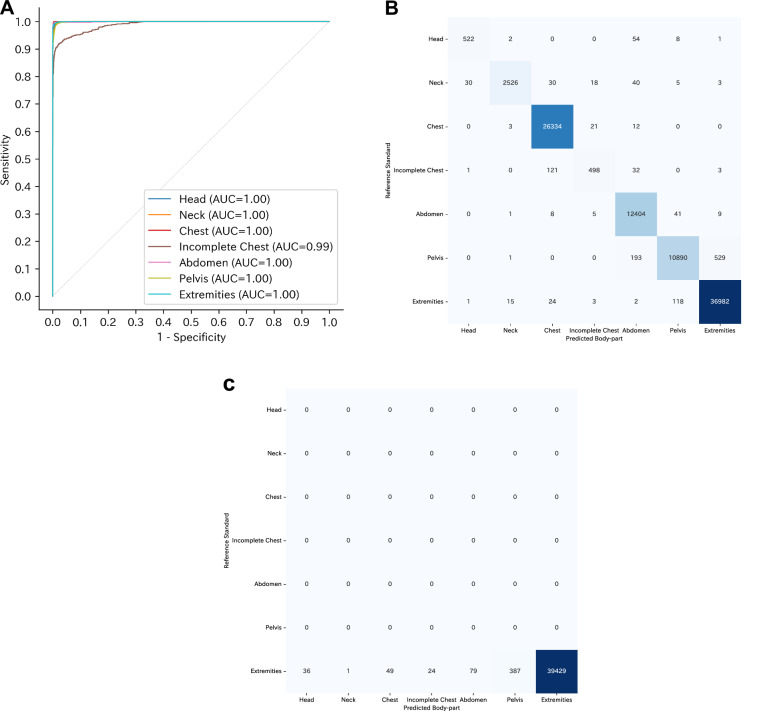
Receiver operating characteristic curves and confusion matrices of Xp-Bodypart-Checker. **A** Receiver operating characteristic curve for body-part classification (seven categories) using the external dataset from Institution B. **B** Confusion matrix for body-part classification in the external dataset from Institution B. **C** Confusion matrix for body-part classification in the external dataset from MURA. For the external dataset from MURA, which contains only “Extremities” radiographs, no receiver operating characteristic curve is shown because there is only one class. AUC, area under the receiver operating characteristic curve

For Xp-Bodypart-Checker, the best-performing model was obtained at 11 epochs with the lowest loss value. The overall model performance was assessed through micro-, macro-, and weighted-accuracy calculations. For the external test set from Institution B, these accuracy metrics were 98.5% (99% CI: 98.4%–98.6%), 99.6% (99% CI: 99.6%–99.6%), and 99.4% (99% CI: 99.4%–99.5%), respectively. Additionally, we computed class-specific accuracy scores for each category; for example, the accuracy for the Chest class was 99.8% (99% CI: 99.7%–99.8%). To assess the model’s discriminative capability, we generated one-vs.-all ROC curves for each class and calculated the corresponding AUC values, which ranged from 0.99 (99% CI: 0.98–0.99) for the Incomplete Chest class to 1.00 (99% CI: 1.00–1.00) for the Head, Neck, Chest, Abdomen, Pelvis, and Extremities classes. The ROC curves are presented in Fig. 4A, and the accuracy and AUC values for both the overall performance and each category are summarised in Table 3. Confusion matrices were created to illustrate performance across all categories (Fig. 4B). For the external test set from MURA, the micro-accuracy was 98.6% (99% CI: 98.4%–98.7%). The accuracy and AUC values for both the overall performance and each category are summarised in Table 3. Confusion matrices were created to illustrate performance across all categories (Fig. 4C). Results of Xp-Bodypart-Checker with mislabeled data from Institution B are shown in the Supplementary appendix (p 8). Results of Xp-Bodypart-Checker on radiographs from Institution B and MURA with side markers removed are shown in the Supplementary appendix (p 9). Results of Xp-Bodypart-Checker based on the ResNet-50 architecture, and results of Xp-Bodypart-Checker based on the HOG feature extraction method with an SVM classifier are shown in the Supplementary appendix (p 10, 11).

**Table 3:** Results of Xp-bodypart-checker

	Institution A		Institution B		MURA	
	Internal test		External test		External test	
Overall						
Micro average accuracy (%)	99.7	(99.6–99.8)	98.5	(98.4–98.6)	98.6	(98.4–98.7)
Macro average accuracy (%)	99.9	(99.6–99.8)	99.6	(99.6–99.6)	N/A	N/A
Weighted average accuracy (%)	99.9	(99.6–99.8)	99.4	(99.4–99.5)	N/A	N/A
Body parts within radiographs						
Head						
Accuracy (%)	> 99.9	(99.9–100.0)	99.9	(99.9–99.9)	N/A	N/A
AUC	1.00	(1.00–1.00)	1.00	(1.00–1.00)	N/A	N/A
Neck						
Accuracy (%)	> 99.9	(99.9–100.0)	99.8	(99.8–99.9)	N/A	N/A
AUC	1.00	(1.00–1.00)	1.00	(1.00–1.00)	N/A	N/A
Chest						
Accuracy (%)	99.8	(99.8–99.9)	99.8	(99.7–99.8)	N/A	N/A
AUC	1.00	(1.00–1.00)	1.00	(1.00–1.00)	N/A	N/A
Incomplete Chest						
Accuracy (%)	99.8	(99.7–99.8)	99.8	(99.7–99.8)	N/A	N/A
AUC	1.00	(1.00–1.00)	0.99	(0.98–0.99)	N/A	N/A
Abdomen						
Accuracy (%)	99.9	(99.8–99.9)	99.6	(99.5–99.6)	N/A	N/A
AUC	1.00	(1.00–1.00)	1.00	(1.00–1.00)	N/A	N/A
Pelvis						
Accuracy (%)	99.9	(99.9–100.0)	99.0	(98.9–99.1)	N/A	N/A
AUC	1.00	(1.00–1.00)	1.00	(1.00–1.00)	N/A	N/A
Extremities						
Accuracy (%)	99.9	(99.9–100.0)	99.2	(99.2–99.3)	98.6	(98.4–98.7)
AUC	1.00	(1.00–1.00)	1.00	(1.00–1.00)	N/A	N/A

Data are percentages (99% confidence interval) unless otherwise stated

*AUC* area under the receiver operating characteristic curve

**Fig. 5:**
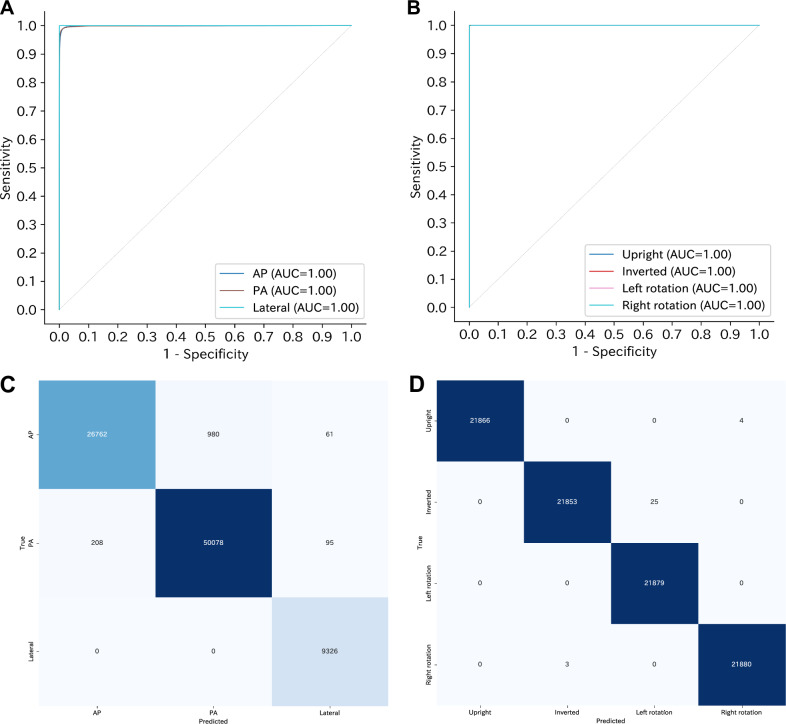
Receiver operating characteristic curves and confusion matrices of CXp-Projection-Rotation-Checker. **A** One-vs.-all receiver operating characteristic curves for projection classification (AP, PA, Lateral) using the external dataset from Institution A. **B** One-vs.-all receiver operating characteristic curves for rotation classification (upright, inverted, left rotation, right rotation) using the external dataset from Institution A. **C** Confusion matrix for projection classification in the same external dataset. **D** Confusion matrix for rotation classification in the same external dataset. AP, anterior-posterior; PA, posterior-anterior; AUC, area under the receiver operating characteristic curve

For CXp-Projection-Rotation-Checker, the best-performing model was obtained at 5 epochs with the lowest loss value. We evaluated micro-, macro-, and weighted-accuracy for both projection and rotation classifications. On the external test set from Institution A, projection classification reached a micro-accuracy of 98.5% (99% CI: 98.4%–98.6%), macro-accuracy of 98.5% (99% CI: 98.4%–98.7%), and a weighted-accuracy of 98.5% (99% CI: 98.4%–98.6%). Rotation classification achieved a micro-accuracy of over 99.9% (99% CI: 99.9%–100.0%), macro-accuracy of over 99.9% (99% CI: 99.9%–100.0%), and a weighted-accuracy of over 99.9% (99% CI: 99.9%–100.0%). Additionally, we computed class-specific accuracy scores for each category within each classification. To assess the model’s discriminative capability, we generated ROC curves and calculated AUC values for each class using a one-vs.-all approach. For projection classification, the AUC values were 1.00 (99% CI: 1.00–1.00) for the AP, PA, and lateral views. For rotation classification, the AUC values were 1.00 (99% CI: 1.00–1.00) for the upright, inverted, left rotation, and right rotation views. The ROC curves are shown in Fig. 5A, B, the confusion matrices are presented in Fig. 5C, D, and the accuracy and AUC values both overall and for each category are presented in Table 4. Results of CXp-Projection-Rotation-Checker with mislabeled data from Institution A are shown in the Supplementary appendix (p 12). Results of CXp-Projection-Rotation-Checker with real rotation labels from Institution A are shown in the Supplementary appendix (p 13). Results of CXp-Projection-Rotation-Checker on radiographs from Institution A with side markers removed are shown in the Supplementary appendix (p 14). Results of CXp-Projection-Rotation-Checker based on the ResNet-50 architecture and Results of CXp-Projection-Rotation-Checker based on the HOG feature extraction method with an SVM classifier are shown in the Supplementary appendix (p 15–16).

**Table 4:** Results of CXp-projection-rotation-checker

	CheXpert		PadChest		Institution A	
	Internal test		Internal test		External test	
Projection						
Overall accuracy						
Micro average accuracy (%)	99.3	(99.1–99.4)	99.0	(98.9–99.2)	98.5	(98.4–98.6)
Macro average accuracy (%)	99.3	(99.1–99.4)	99.0	(98.9–99.2)	98.5	(98.4–98.7)
Weighted average accuracy (%)	99.3	(99.1–99.4)	99.0	(98.8–99.2)	98.5	(98.4–98.6)
Frontal						
AP						
Accuracy (%)	99.3	(99.1–99.4)	99.5	(99.4–99.7)	98.6	(98.5–98.7)
AUC	1.00	(1.00–1.00)	1.00	(0.99–1.00)	1.00	(1.00–1.00)
PA						
Accuracy (%)	99.3	(99.1–99.4)	99.1	(98.9–99.3)	98.5	(98.4–98.6)
AUC	1.00	(1.00–1.00)	1.00	(1.00–1.00)	1.00	(1.00–1.00)
Lateral						
Accuracy (%)	> 99.9	(99.9–100.0)	99.5	(99.3–99.6)	99.8	(99.8–99.9)
AUC	1.00	(1.00–1.00)	1.00	(1.00–1.00)	1.00	(1.00–1.00)
Rotation						
Overall accuracy						
Micro average accuracy (%)	99.9	(99.9–100.0)	99.5	(99.4–99.7)	> 99.9	(99.9–100.0)
Macro average accuracy (%)	99.9	(99.9–100.0)	99.5	(99.4–99.7)	> 99.9	(99.9–100.0)
Weighted average accuracy (%)	99.9	(99.9–100.0)	99.5	(99.4–99.7)	> 99.9	(99.9–100.0)
Upright						
Accuracy (%)	> 99.9	(99.9–100.0)	99.8	(99.7–99.9)	> 99.9	(> 99.9–100.0)
AUC	1.00	(1.00–1.00)	1.00	(1.00–1.00)	1.00	(1.00–1.00)
Inverted						
Accuracy (%)	> 99.9	(99.9–1.00)	99.8	(99.7–99.9)	> 99.9	(> 99.9–100.0)
AUC	1.00	(1.00–1.00)	1.00	(1.00–1.00)	1.00	(1.00–1.00)
Left rotation						
Accuracy (%)	> 99.9	(99.9–1.00)	99.8	(99.7–99.9)	> 99.9	(99.9–1.00)
AUC	1.00	(1.00–1.00)	1.00	(1.00–1.00)	1.00	(1.00–1.00)
Right rotation						
Accuracy (%)	99.9	(99.9–1.00)	99.7	(99.6–99.8)	> 99.9	(99.9–1.00)
AUC	1.00	(1.00–1.00)	1.00	(1.00–1.00)	1.00	(1.00–1.00)

Data are percentages (99% confidence interval) unless otherwise stated

*AP* anterior-posterior, *PA* posterior-anterior, *AUC* area under the receiver operating characteristic curve

**Fig. 6:**
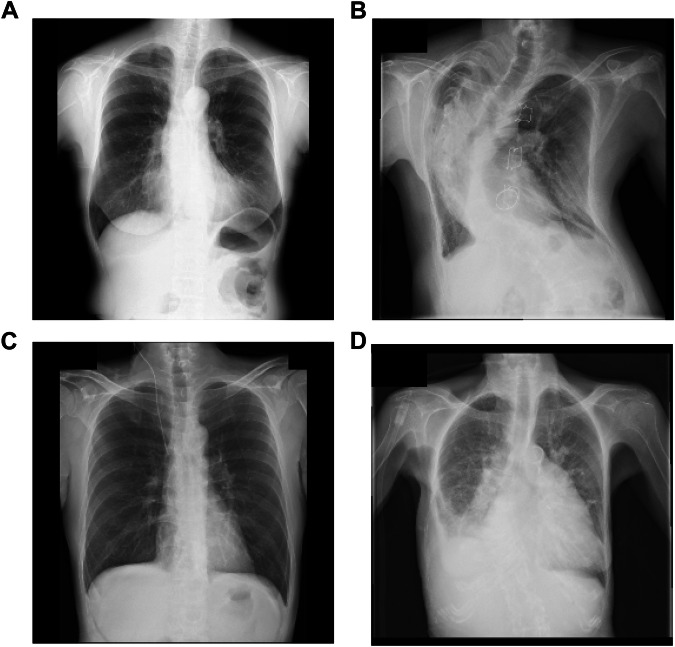
Representative examples of misclassifications by Xp-Bodypart-Checker and CXp-Projection-Rotation-Checker. **A** Chest radiograph of a female in her 50 s that was misclassified as “Abdomen” by Xp-Bodypart-Checker. **B** PA chest radiograph of a female in her 40 s that was misclassified as “Lateral” by CXp-Projection-Rotation-Checker. **C** AP chest radiograph of a male in his 50 s that was misclassified as “PA” by CXp-Projection-Rotation-Checker. **D** PA chest radiograph of a female in her 80 s that was misclassified as “AP” by CXp-Projection-Rotation-Checker. AP, anterior-posterior; PA, posterior-anterior; AUC, area under the receiver operating characteristic curve

In addition, to establish the characteristics of the misclassified images, we selected representative examples from the external test sets where the models’ predictions were incorrect. The example for Xp-Bodypart-Checker is shown in Fig. 6A. These examples for CXp-Projection-Rotation-Checker are shown in Fig. 6B, C, D.

Saliency maps are shown in the Supplementary appendix (p 5, 6).

We developed and deployed web applications that leverage Xp‑Bodypart‑Checker and CXp‑Projection‑Rotation‑Checker to compute confidence scores and detect mislabeled images (https://huggingface.co/spaces/MedicalAILabo/Xp-Bodypart-Mislabel-Checker, https://huggingface.co/spaces/MedicalAILabo/CXp-Projection-Rotation-Mislabel-Checker). A screenshot of the application in use is provided in the Supplementary appendix (p 7).

## Discussion

In this study, we developed two deep-learning models—Xp-Bodypart-Checker and CXp-Projection-Rotation-Checker—to address metadata inconsistencies in large-scale radiograph datasets. Xp-Bodypart-Checker was trained, tuned, and internally tested on data from Institution A and externally tested using data from Institution B and MURA. CXp-Projection-Rotation-Checker was trained, tuned, and internally tested using data from CheXpert and PadChest, then externally tested using data from Institution A. Altogether, over 400,000 radiographs were involved in developing Xp-Bodypart-Checker, and over 460,000 chest radiographs were involved for CXp-Projection-Rotation-Checker. Xp-Bodypart-Checker classified radiographs into seven categories (Head, Neck, Chest, Incomplete Chest, Abdomen, Pelvis, and Extremities) with excellent performance, achieving AUC values higher than 0.99. Similarly, CXp-Projection-Rotation-Checker distinguished chest radiograph projection (AP, PA, and Lateral) and rotation (upright, inverted, left rotation, or right rotation) with AUC values of 1.00.

The primary strength of our study is the development and external test of two high‑performance radiograph‑classification models using an unprecedentedly large, multi‑centre dataset. Xp‑Bodypart‑Checker expands conventional body‑part classifiers by discriminating chest radiographs in which the lung fields are fully versus only partially visible. CXp‑Projection‑Rotation‑Checker simultaneously classifies imaging projection and rotation within a single network. Previous work has usually tackled only one of these subtasks—body‑part recognition [10, 12, 19] or projection/rotation identification [13–18, 20, 21, 31]—and has relied on smaller, often single‑institution cohorts. By aggregating images from multiple institutions with heterogeneous acquisition protocols, we enhanced the generalizability of our models. On an external test set from Institution B, Xp‑Bodypart‑Checker achieved an accuracy of 98.5%, exceeding the 86–97.4% range reported in earlier studies [10, 12]. Likewise, CXp‑Projection‑Rotation‑Checker, trained on 463,728 chest radiographs from several countries, obtained accuracies of 98.5% for projection and 99.3% for rotation on external data from Institution A. These results equal or surpass the best published figures for projection (96 to > 99%) and rotation (88 to > 99%) classification [13–18, 31], demonstrating that integrating both subtasks in a single architecture can deliver state‑of‑the‑art performance across diverse clinical settings.

Accurate DICOM labeling is important for both clinical settings and large-scale research endeavors. In our study, 3.9% of radiographs at Institution A and 12.8% at Institution B required body-part label corrections, while 3.3% of chest radiographs at Institution A had incorrect projection tags. In vast imaging archives, even minor error rates quickly add up to hundreds or thousands of mislabeled examinations. Clinically, such errors can delay patient care—for instance, mislabeling an “AP” projection as “PA” may affect orientation-based measurements [32]. In research pipelines, incorrect labels can compromise DL training [33, 34]. By automating the detection of mislabeled studies, our models help ensure data integrity at the front end of both clinical workflows and research pipelines. We have developed and publicly released web applications that leverage Xp‑Bodypart‑Checker and CXp‑Projection‑Rotation‑Checker to detect mislabeled radiographic images (https://huggingface.co/spaces/MedicalAILabo/Xp-Bodypart-Mislabel-Checker, https://huggingface.co/spaces/MedicalAILabo/CXp-Projection-Rotation-Mislabel-Checker). By integrating this capability into the Picture Archiving and Communication System, users can manually review any images that trigger a warning, enabling correction of mislabeled cases with minimal effort. Furthermore, we plan to retrain the model on radiographs that were flagged despite being correctly labeled, as well as those that were not flagged but were in fact mislabeled, to achieve even greater accuracy. This not only saves time and resources but also underpins safer clinical decisions and more trustworthy scientific findings.

Despite the high overall accuracy, we found interesting results and areas for improvement through error analysis. Xp-Bodypart-Checker sometimes mislabeled chest radiographs that included the upper abdomen as “Abdomen.” Overlapping structures, such as the diaphragm and lower thorax, can blur the boundary between the chest and abdomen, suggesting the need for more flexible labeling or continuous scores in borderline cases. Similarly, CXp-Projection-Rotation-Checker struggled with certain anatomical variations. We observed that chest radiographs with a noticeably large cardiac silhouette in PA views were occasionally misclassified as AP, while those with a smaller cardiac silhouette in AP views were sometimes mistaken for PA. This likely reflects the known tendency for AP images to exaggerate heart size [32]. In addition, PA chest radiographs showing marked spinal scoliosis were at times confused with lateral views, implying that the model may heavily rely on spine orientation cues. These findings indicate that training on more diverse cases and incorporating additional clinical context—such as patient posture or spinal alignment—could further enhance reliability.

This study had several limitations. First, the study did not include certain populations (e.g., paediatric patients) or full-body scans that include both trunk and extremities, limiting immediate relevance to those settings. Second, institution-specific labeling protocols might introduce biases, and integrating these models into established systems could require technical adjustments.

Our two-model strategy provides a practical way to detect and fix common labeling errors in large radiographic collections. By automatically verifying body-part, projection, and rotation tags, it lays a stronger groundwork for routine clinical tasks and research projects. Future efforts will involve real-time testing, applications in paediatric or more complex imaging scenarios, and possible multi-label outputs for borderline cases. We believe that robust, automated labeling is a key step toward more reliable and versatile radiographic databases.

## Supplementary information

Supplementary information

## Data Availability

Xp-Bodypart-Checker and CXp-Projection-Rotation-Checker are available on Hugging Face (https://huggingface.co/spaces/MedicalAILabo/Xp-Bodypart-Checker, https://huggingface.co/spaces/MedicalAILabo/CXp-Projection-Rotation-Checker). Additionally, web applications have been launched on Hugging Face that identify and flag mislabeled images based on their assigned labels and the model’s predicted probabilities (https://huggingface.co/spaces/MedicalAILabo/Xp-Bodypart-Mislabel-Checker, https://huggingface.co/spaces/MedicalAILabo/CXp-Projection-Rotation-Mislabel-Checker). However, chest radiographs from Institutions A and B are not available at this time, as these have been withheld by the hospitals that participated in the trials to protect participant privacy. Additionally, data from CheXpert, PadChest, and MURA were used for this study; these datasets are accessible through their respective repositories (see References 23, 24, and 25).

## Abbreviations

AP: Anterior-posterior
AUC: Area under the receiver operating characteristic curve
DL: Deep learning
HOG: Histogram of oriented gradients
PA: Posterior-anterior
