# Supplementary material for: Deep learning models for radiography body-part classification and chest radiograph projection/orientation classification: a multi-institutional study
Source: Eur Radiol. 2025 Oct 22;36(4):2576–90. doi: 10.1007/s00330-025-12053-7 (PMC13035548; doi:10.1007/s00330-025-12053-7)
Supplement: Supplementary file 1 — Supplementary information [file 330_2025_12053_MOESM1_ESM.pdf]

Supplementary Appendix

Deep Learning Models for Radiography Body-part Classification and Chest Radiograph Projection/Orientation Classification: A Multi-institutional Study

Authors:
Yasuhito Mitsuyama MD, Hirotaka Takita MD, PhD, Shannon L Walston PhD, Ko Watanabe PhD, Shoya Ishimaru PhD, Yukio Miki MD, PhD, Daiju Ueda MD, PhD

Table of Contents:

Section S1: Supplementary Methods
1) Detailed process for parameter tuning of the deep learning model
2) Detailed process for parameter tuning of the deep learning model using ResNet-50
3) Detailed process for parameter tuning of the deep learning model using Histogram of Oriented Gradients feature extraction with a linear Support Vector Machine classifier
4) Machine environment

Section S2: Supplementary Figures
Appendix Figure 1: Changes in body-part label after two radiologists' review
Appendix Figure 2: Changes in projection label after two radiologists' review
Appendix Figure 3: Saliency maps of Xp-Bodypart-Checker using external test datasets from institution B
Appendix Figure 4: Saliency maps of CXp-Projection-Rotation-Checker using external test datasets from institution A
Appendix Figure 5: Web application to detect mislabeled radiographic images

Section S3: Supplementary Tables
Appendix Table 1: Results of Xp-Bodypart-Checker with mislabeled datasets from Institution B
Appendix Table 2: Results of Xp-Bodypart-Checker on radiographs from Institution B and MURA with side markers removed
Appendix Table 3: Results of Xp-Bodypart-Checker based on ResNet-50
Appendix Table 4: Results of Xp-Bodypart-Checker based on Histogram of Oriented Gradients feature extraction with a Support Vector Machine classifier
Appendix Table 5: Results of CXp-Projection-Rotation-Checker with mislabeled datasets from Institution A
Appendix Table 6: Results of CXp-Projection-Rotation-Checker with real rotation label dataset from Institution A
Appendix Table 7: Results of CXp-Projection-Rotation-Checker on radiographs from Institution A with side markers removed
Appendix Table 8: Results of CXp-Projection-Rotation-Checker based on ResNet-50
Appendix Table 9: Results of CXp-Projection-Rotation-Checker based on Histogram of Oriented Gradients feature extraction with a Support Vector Machine classifier

Section S4: References for the Supplementary Appendix

Section S1: Methods

1) Detailed process for parameter tuning of the deep learning model

In this study, we developed two deep learning models, Xp-Bodypart-Checker and CXp-Projection-Rotation-Checker. Both models were built on the EfficientNetB4 architecture. For each model, we performed hyperparameter tuning for the optimizer, learning rate, and batch size. Specifically, we employed Adam as the optimizer for both models (the learning rate was searched within 0.001–0.05) and a batch size of 32 was used.

2) Detailed process for parameter tuning of the deep learning model using ResNet-50

For benchmarking, we developed ResNet-50 models under an identical data split, preprocessing pipeline, augmentation strategy, and optimization schedule; the only difference was replacement of the EfficientNet backbone by ResNet-50. For each model, we performed hyperparameter tuning for the optimizer, learning rate, and batch size. Specifically, we employed Adam as the optimizer for both models (the learning rate was searched within 0.001–0.05) and a batch size of 32 was used.

3) Detailed process for parameter tuning of the deep learning model using Histogram of Oriented Gradients feature extraction with a linear Support Vector Machine classifier

Classical computer-vision baselines were constructed by extracting Histogram of Oriented Gradients descriptors from the same 256 × 256 pixel, 8-bit greyscale images. We used 9 unsigned orientation bins, square cells of 16 × 16 pixels, and blocks comprising 2 × 2 cells (block stride: one cell); block vectors were normalised with the L2-Hys scheme, yielding 8 100-dimensional feature vectors per image. These feature vectors were used without further standardisation as input to a linear Support Vector Machine implemented via scikit-learn's SGDClassifier (loss="hinge", learning_rate="optimal", random_state=42) with class_weight="balanced" (inverse frequency weighting). Regularisation strength α was tuned over $\{1 \times 10^{-4}, 1 \times 10^{-3}, 1 \times 10^{-2}\}$ together with Elastic-Net mixing ratio l1_ratio $\in \{0, 0.15\}$ by grid search, selecting the combination that maximised balanced accuracy on a held-out validation set. Model training proceeded for up to 100 epochs via partial_fit on a randomly permuted training set each epoch.

4) Machine environment

We adopted Ubuntu 20.04 (Canonical, London, England) with the PyTorch deep learning framework (version 2.0.1; The Linux Foundation; https://pytorch.org), with CUDA 11.8 (Nvidia Corporation, Santa Clara, CA) dependencies for graphics processing unit acceleration. We used a GDEP Advance Deep Learning Box equipped with four NVIDIA Titan V graphics processing units (Nvidia Corporation).

Section S2: Supplementary Figures

Appendix Figure 1: Changes in body-part label before and after two radiologists' review

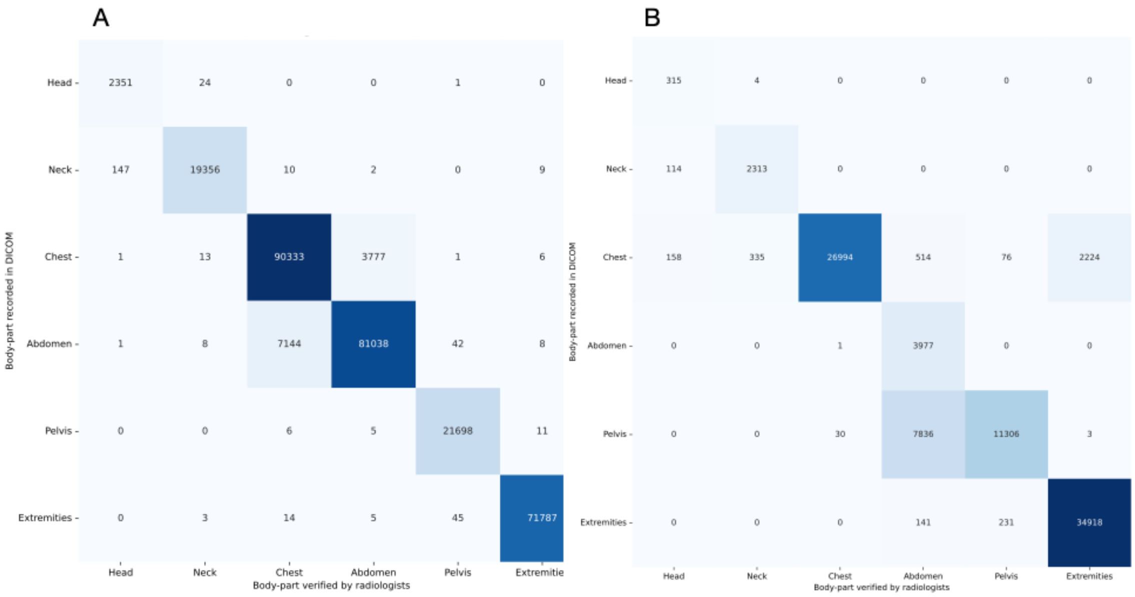

(A) Changes in body-part label of radiographs from Institution A before and after two radiologists' review. (B) Changes in body-part label of radiographs from Institution B before and after two radiologists' review.

The rows list the original body-part labels from the DICOM metadata, and the columns list the body-part labels assigned after verification by two board-certified radiologists. Each cell shows how many radiographs were confirmed or corrected. The diagonal cells where the row and column labels match represent the number of radiographs where the radiologists agreed with the original DICOM labels, while the off-diagonal cells show the number of radiographs that were changed.

Appendix Figure 2: Changes in projection label after two radiologists' review

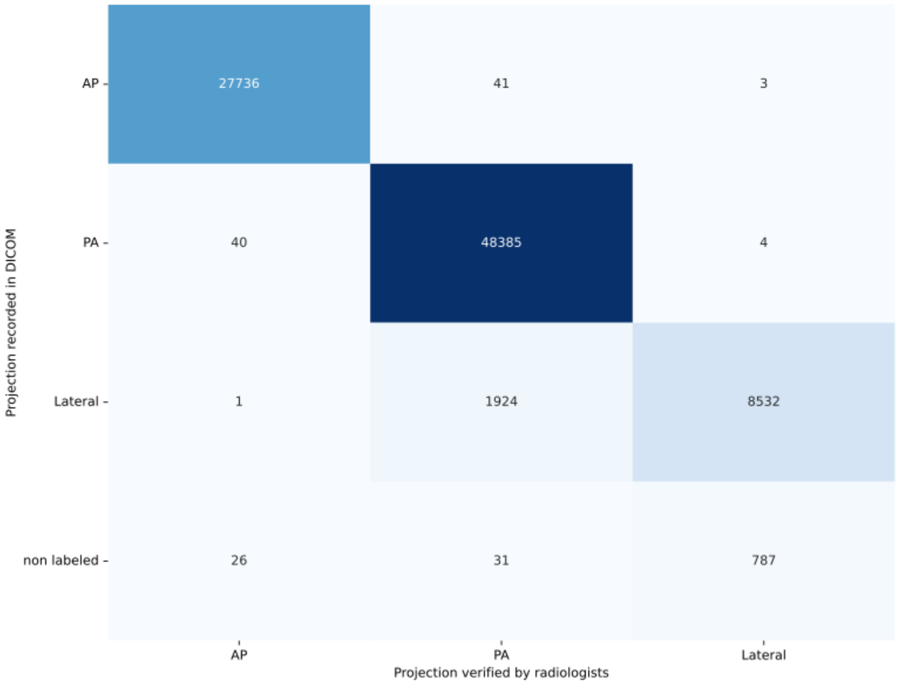

Changes in projection label of chest radiographs from Institution A before and after two radiologists' review.
The rows list the original projection labels from the DICOM metadata, and the columns list the projection labels assigned after verification by two board-certified radiologists. Each cell shows how many chest radiographs were confirmed or corrected. The diagonal cells where the row and column labels match represent the number of chest radiographs where the radiologists agreed with the original DICOM labels, while the off-diagonal cells show the number of chest radiographs that were changed.

Appendix Figure 3: Saliency maps of Xp-Bodypart-Checker using external test datasets from institution B

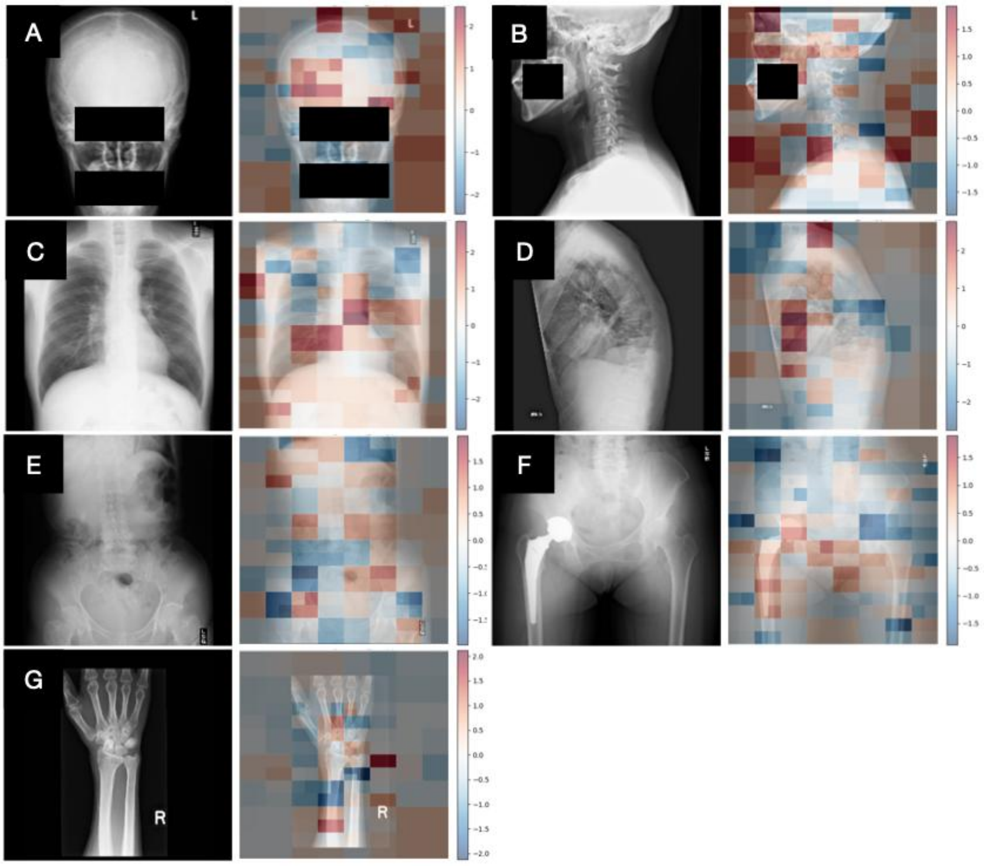

(A) Saliency map of a radiograph correctly classified as “Head” by Xp-Bodypart-Checker. (B) Saliency map of a radiograph correctly classified as “Neck” by Xp-Bodypart-Checker. (C) Saliency map of a radiograph correctly classified as “Chest” by Xp-Bodypart-Checker. (D) Saliency map of a radiograph correctly classified as “Incomplete Chest” by Xp-Bodypart-Checker. (E) Saliency map of a radiograph correctly classified as “Abdomen” by Xp-Bodypart-Checker. (F) Saliency map of a radiograph correctly classified as “Pelvis” by Xp-Bodypart-Checker. (G) Saliency map of a radiograph correctly classified as “Extremities” by Xp-Bodypart-Checker.

Appendix Figure 4: Saliency maps of CXp-Projection-Rotation-Checker using external test datasets from institution A

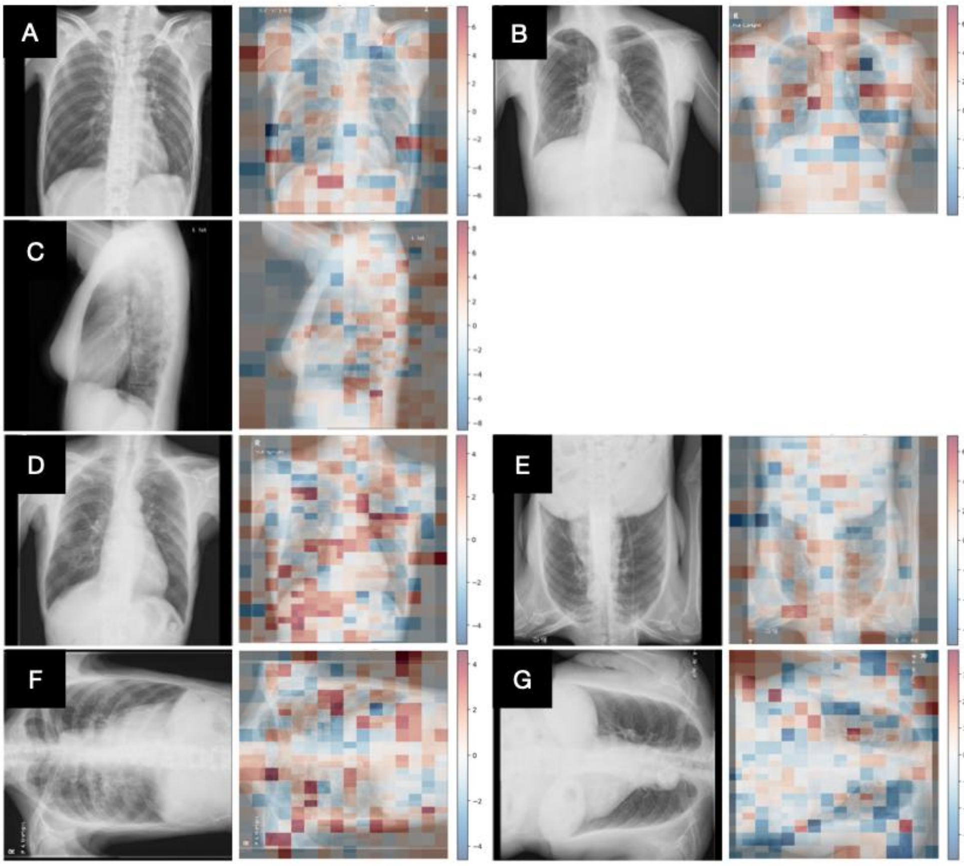

(A) Saliency map of a chest radiograph correctly classified as "AP" by CXp-Projection-Rotation-Checker. (B) Saliency map of a chest radiograph correctly classified as "PA" by CXp-Projection-Rotation-Checker. (C) Saliency map of a chest radiograph correctly classified as "Lateral" by CXp-Projection-Rotation-Checker. (D) Saliency map of a chest radiograph correctly classified as "Upright" by CXp-Projection-Rotation-Checker. (E) Saliency map of a chest radiograph correctly classified as "Inverted" by CXp-Projection-Rotation-Checker. (F) Saliency map of a chest radiograph correctly classified as "Left rotation" by CXp-Projection-Rotation-Checker. (G) Saliency map of a chest radiograph correctly classified as "Right rotation" by CXp-Projection-Rotation-Checker.

Appendix Figure 5: Web application to detect mislabeled radiographic images

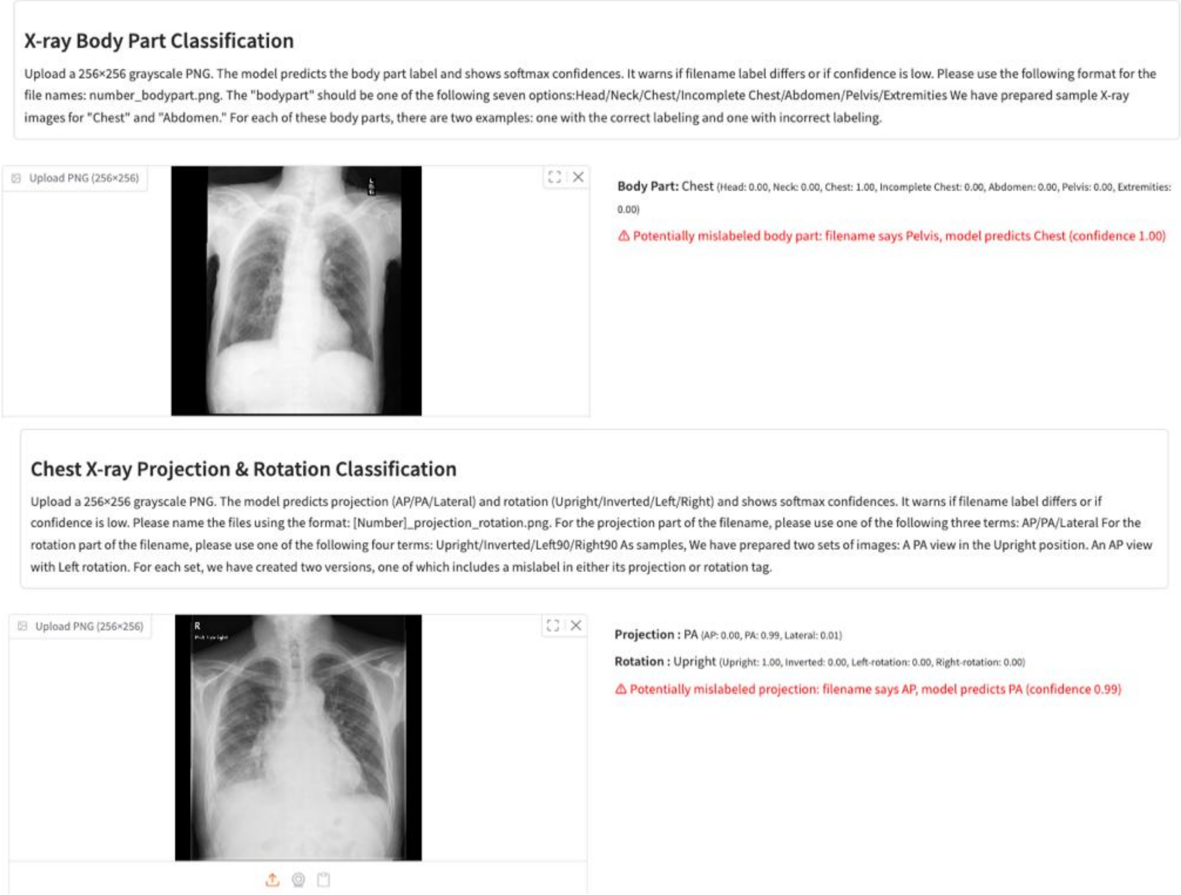

A screenshot of applications that compare the label attached to a radiographic image with the label and confidence score predicted by Xp-Bodypart-Checker or CXp-Projection-Rotation-Checker, and display a warning if there’s a risk of mislabelling.

Section S3: Supplementary Tables

Appendix Table 1: Results of Xp-Bodypart-Checker with mislabeled datasets from Institution B

	Institution B	
	External test	
Overall		
Micro average accuracy (%)	96.1	(95.6–96.6)
Macro average accuracy (%)	98.9	(98.7–99.0)
Weighted average accuracy (%)	98.3	(98.1–98.6)
Body-part within radiographs		
Head		
Accuracy (%)	99.2	(99.0–99.4)
AUC	1.00	(1.00–1.00)
Neck		
Accuracy (%)	98.8	(98.5–99.0)
AUC	0.99	(0.98–0.99)
Chest		
Accuracy (%)	99.4	(99.2–99.6)
AUC	1.00	(0.99–1.00)
Incomplete Chest		
Accuracy (%)	99.7	(99.5–99.8)
AUC	0.96	(0.88–1.00)
Abdomen		
Accuracy (%)	98.2	(97.8–98.4)
AUC	1.00	(1.00–1.00)
Pelvis		
Accuracy (%)	98.3	(98.0–98.7)
AUC	0.99	(0.99–1.00)
Extremities		
Accuracy (%)	98.7	(98.4–99.0)
AUC	1.00	(1.00–1.00)

Data are percentages (99% confidence interval) unless otherwise stated. AUC=area under the receiver operating characteristic curve.

Appendix Table 2: Results of Xp-Bodypart-Checker on radiographs from Institution B and MURA with side markers removed

	Institution B		MURA	
	External test		External test	
Overall				
Micro average accuracy (%)	98.5	(98.4–98.6)	98.6	(98.5–98.8)
Macro average accuracy (%)	99.6	(99.5–99.6)	N/A	N/A
Weighted average accuracy (%)	99.4	(99.4–99.5)	N/A	N/A
Body-part within radiographs				
Head				
Accuracy (%)	99.9	(99.9–99.9)	N/A	N/A
AUC	1.00	(1.00–1.00)	N/A	N/A
Neck				
Accuracy (%)	99.8	(99.8–99.9)	N/A	N/A
AUC	1.00	(1.00–1.00)	N/A	N/A
Chest				
Accuracy (%)	99.7	(99.7–99.8)	N/A	N/A
AUC	1.00	(1.00–1.00)	N/A	N/A
Incomplete Chest				
Accuracy (%)	99.7	(99.6–99.7)	N/A	N/A
AUC	0.98	(0.97–0.99)	N/A	N/A
Abdomen				
Accuracy (%)	99.6	(99.5–99.7)	N/A	N/A
AUC	1.00	(1.00–1.00)	N/A	N/A
Pelvis				
Accuracy (%)	99.0	(99.0–99.1)	N/A	N/A
AUC	1.00	(1.00–1.00)	N/A	N/A
Extremities				
Accuracy (%)	99.2	(99.1–99.3)	98.6	(98.5–98.8)
AUC	1.00	(1.00–1.00)	N/A	N/A

Data are percentages (99% confidence interval) unless otherwise stated. AUC=area under the receiver operating characteristic curve.

Appendix Table 3: Results of Xp-Bodypart-Checker based on ResNet-50

	Institution A		Institution B		MURA	
	Internal test		External test		External test	
Overall						
Micro average accuracy (%)	99.7	(99.6–99.8)	98.3	(98.2–98.4)	97.5	(97.3–97.7)
Macro average accuracy (%)	99.9	(99.9–99.9)	99.5	(99.5–99.5)	N/A	N/A
Weighted average accuracy (%)	99.9	(99.9–99.9)	99.4	(99.3–99.4)	N/A	N/A
Body-part within radiographs						
Head						
Accuracy (%)	>99.9	(99.9–100.0)	99.8	(99.8–99.9)	N/A	N/A
AUC	1.00	(1.00–1.00)	1.00	(1.00–1.00)	N/A	N/A
Neck						
Accuracy (%)	>99.9	(99.9–100.0)	99.7	(99.7–99.8)	N/A	N/A
AUC	1.00	(1.00–1.00)	1.00	(0.99–1.00)	N/A	N/A
Chest						
Accuracy (%)	99.8	(99.8–99.9)	99.7	(99.7–99.8)	N/A	N/A
AUC	1.00	(1.00–1.00)	1.00	(1.00–1.00)	N/A	N/A
Incomplete Chest						
Accuracy (%)	99.8	(99.7–99.9)	99.7	(99.6–99.7)	N/A	N/A
AUC	1.00	(1.00–1.00)	0.98	(0.98–0.99)	N/A	N/A
Abdomen						
Accuracy (%)	99.9	(99.8–99.9)	99.5	(99.4–99.5)	N/A	N/A
AUC	1.00	(1.00–1.00)	1.00	(1.00–1.00)	N/A	N/A
Pelvis						
Accuracy (%)	99.9	(99.9–100.0)	99.0	(98.9–99.1)	N/A	N/A
AUC	1.00	(1.00–1.00)	1.00	(1.00–1.00)	N/A	N/A
Extremities						
Accuracy (%)	>99.9	(99.9–100.0)	99.2	(99.1–99.2)	97.5	(97.3–97.7)
AUC	1.00	(1.00–1.00)	1.00	(1.00–1.00)	N/A	N/A

Data are percentages (99% confidence interval) unless otherwise stated. AUC=area under the receiver operating characteristic curve.

Appendix Table 4: Results of Xp-Bodypart-Checker based on Histogram of Oriented Gradients feature extraction with a Support Vector Machine classifier

	Institution A		Institution B		MURA	
	Internal test		External test		External test	
Overall						
Micro average accuracy (%)	99.2	(99.0–99.3)	93.1	(92.9–93.3)	88.8	(88.4–89.2)
Macro average accuracy (%)	99.8	(99.7–99.8)	98.0	(98.0–98.1)	N/A	N/A
Weighted average accuracy (%)	99.7	(99.6–99.8)	97.6	(97.5–97.7)	N/A	N/A
Body-part within radiographs						
Head						
Accuracy (%)	>99.9	(99.9–100.0)	99.1	(99.0–99.1)	N/A	N/A
AUC	1.00	(1.00–1.00)	0.89	(0.87–0.91)	N/A	N/A
Neck						
Accuracy (%)	99.9	(99.9–100.0)	97.9	(97.8–98.0)	N/A	N/A
AUC	1.00	(1.00–1.00)	0.97	(0.97–0.98)	N/A	N/A
Chest						
Accuracy (%)	99.6	(99.5–99.7)	98.6	(98.5–98.7)	N/A	N/A
AUC	1.00	(1.00–1.00)	1.00	(1.00–1.00)	N/A	N/A
Incomplete Chest						
Accuracy (%)	99.6	(99.5–99.7)	99.1	(99.0–99.2)	N/A	N/A
AUC	1.00	(0.99–1.00)	0.97	(0.96–0.98)	N/A	N/A
Abdomen						
Accuracy (%)	99.7	(99.6–99.8)	98.0	(97.8–98.1)	N/A	N/A
AUC	1.00	(1.00–1.00)	0.99	(0.99–1.00)	N/A	N/A
Pelvis						
Accuracy (%)	99.8	(99.7–99.9)	96.6	(96.4–96.8)	N/A	N/A
AUC	1.00	(1.00–1.00)	0.98	(0.97–0.98)	N/A	N/A
Extremities						
Accuracy (%)	99.7	(99.7–99.8)	97.0	(96.8–97.1)	88.8	(88.4–89.2)
AUC	1.00	(1.00–1.00)	0.99	(0.99–0.99)	N/A	N/A

Data are percentages (99% confidence interval) unless otherwise stated. AUC=area under the receiver operating characteristic curve.

Appendix Table 5: Results of CXp-Projection-Rotation-Checker with mislabeled datasets from Institution A

	Institution A	
	External test	
Projection		
Overall Accuracy		
Micro average accuracy (%)	96.6	(95.8–97.5)
Macro average accuracy (%)	97.8	(97.2–98.3)
Weighted average accuracy (%)	96.6	(95.8–97.5)
Frontal		
AP		
Accuracy (%)	96.8	(96.0–97.7)
AUC	0.70	(0.60–0.80)
PA		
Accuracy (%)	98.8	(95.8–97.5)
AUC	0.97	(0.96–0.98)
Lateral		
Accuracy (%)	>99.9	(99.9–100.0)
AUC	1.00	(1.00–1.00)
Rotation		
Overall Accuracy		
Micro average accuracy (%)	99.8	(99.5–100.0)
Macro average accuracy (%)	99.9	(99.8–100.0)
Weighted average accuracy (%)	99.8	(99.5–100.0)
Upright		
Accuracy (%)	99.9	(99.7–100.0)
AUC	1.00	(1.00–1.00)
Inverted		
Accuracy (%)	99.9	(99.7–100.0)
AUC	1.00	(1.00–1.00)
Left rotation		
Accuracy (%)	>99.9	(99.7–100.0)
AUC	1.00	(1.00–1.00)
Right rotation		
Accuracy (%)	99.8	(99.6–100.0)
AUC	1.00	(1.00–1.00)

Data are percentages (99% confidence interval) unless otherwise stated. AP=Anterior-Posterior, PA=Posterior-Anterior. AUC=area under the receiver operating characteristic curve.

Appendix Table 6: Results of CXp-Projection-Rotation-Checker with real rotation label dataset from Institution A

	Institution A	
	External test	
Projection		
Overall Accuracy		
Micro average accuracy (%)	98.8	(98.7–98.9)
Macro average accuracy (%)	99.1	(99.0–99.2)
Weighted average accuracy (%)	98.8	(98.7–98.9)
Frontal		
AP		
Accuracy (%)	98.9	(98.8–99.0)
AUC	1.00	(1.00–1.00)
PA		
Accuracy (%)	98.8	(98.7–98.9)
AUC	1.00	(1.00–1.00)
Lateral		
Accuracy (%)	99.9	(99.8–99.9)
AUC	1.00	(1.00–1.00)
Rotation		
Overall Accuracy		
Micro average accuracy (%)	>99.9	(99.9–100.0)
Macro average accuracy (%)	>99.9	(99.9–100.0)
Weighted average accuracy (%)	>99.9	(99.9–100.0)
Upright		
Accuracy (%)	>99.9	(99.9–100.0)
AUC	N/A	N/A
Inverted		
Accuracy (%)	N/A	N/A
AUC	N/A	N/A
Left rotation		
Accuracy (%)	N/A	N/A
AUC	N/A	N/A
Right rotation		
Accuracy (%)	N/A	N/A
AUC	N/A	N/A

Data are percentages (99% confidence interval) unless otherwise stated. AP=Anterior-Posterior, PA=Posterior-Anterior. AUC=area under the receiver operating characteristic curve.

Appendix Table 7: Results of CXp-Projection-Rotation-Checker on radiographs from Institution A with side markers removed

	Institution A	
	External test	
Projection		
Overall Accuracy		
Micro average accuracy (%)	95.8	(95.6–95.9)
Macro average accuracy (%)	96.1	(96.0–96.3)
Weighted average accuracy (%)	95.8	(95.6–95.9)
Frontal		
AP		
Accuracy (%)	96.6	(96.5–96.8)
AUC	1.00	(1.00–1.00)
PA		
Accuracy (%)	96.0	(95.9–96.2)
AUC	1.00	(1.00–1.00)
Lateral		
Accuracy (%)	98.9	(98.8–99.0)
AUC	1.00	(1.00–1.00)
Rotation		
Overall Accuracy		
Micro average accuracy (%)	>99.9	(99.9–100.0)
Macro average accuracy (%)	>99.9	(99.9–100.0)
Weighted average accuracy (%)	>99.9	(99.9–100.0)
Upright		
Accuracy (%)	>99.9	(99.9–100.0)
AUC	1.00	(1.00–1.00)
Inverted		
Accuracy (%)	>99.9	(99.9–100.0)
AUC	1.00	(1.00–1.00)
Left rotation		
Accuracy (%)	>99.9	(99.9–100.0)
AUC	1.00	(1.00–1.00)
Right rotation		
Accuracy (%)	>99.9	(99.9–100.0)
AUC	1.00	(1.00–1.00)

Data are percentages (99% confidence interval) unless otherwise stated. AP=Anterior-Posterior, PA=Posterior-Anterior. AUC=area under the receiver operating characteristic curve.

Appendix Table 8: Results of CXp-Projection-Rotation-Checker based on ResNet-50

	CheXpert		PadChest		Institution A	
	Internal test		Internal test		External test	
Projection						
Overall Accuracy						
Micro average accuracy (%)	99.3	(99.1–99.4)	90.8	(90.2–91.5)	93.8	(93.6–94.1)
Macro average accuracy (%)	99.3	(99.1–99.4)	90.8	(90.3–91.4)	94.4	(94.2–94.6)
Weighted average accuracy (%)	99.3	(99.1–99.4)	90.8	(90.2–91.4)	93.8	(93.6–94.1)
Frontal						
AP						
Accuracy (%)	99.3	(99.1–99.4)	96.0	(95.6–96.4)	93.8	(93.6–94.1)
AUC	1.00	(1.00–1.00)	0.97	(0.97–0.98)	0.98	(0.98–0.98)
PA						
Accuracy (%)	99.3	(99.1–99.4)	91.8	(91.2–92.3)	93.9	(93.7–94.1)
AUC	1.00	(0.99–1.00)	0.98	(0.98–0.98)	0.98	(0.98–0.98)
Lateral						
Accuracy (%)	>99.9	(99.9–100.0)	93.8	(93.3–94.3)	>99.9	(99.9–100.0)
AUC	1.00	(1.00–1.00)	0.99	(0.99–0.99)	1.00	(1.00–1.00)
Rotation						
Overall Accuracy						
Micro average accuracy (%)	99.9	(99.8–99.9)	93.8	(93.3–94.3)	99.9	(99.8–99.9)
Macro average accuracy (%)	99.9	(99.8–99.9)	93.8	(93.3–94.3)	99.9	(99.8–99.9)
Weighted average accuracy (%)	99.9	(99.8–99.9)	93.8	(93.3–94.3)	99.9	(99.8–99.9)
Upright						
Accuracy (%)	99.9	(99.9–1.00)	97.0	(96.6–97.4)	99.9	(99.8–99.9)
AUC	1.00	(1.00–1.00)	1.00	(1.00–1.00)	1.00	(1.00–1.00)
Inverted						
Accuracy (%)	99.9	(99.9–1.00)	96.7	(96.3–97.0)	99.9	(99.8–99.9)
AUC	1.00	(1.00–1.00)	1.00	(1.00–1.00)	1.00	(1.00–1.00)
Left rotation						
Accuracy (%)	99.9	(99.9–1.00)	96.7	(96.3–97.1)	>99.9	(99.9–1.00)
AUC	1.00	(1.00–1.00)	1.00	(1.00–1.00)	1.00	(1.00–1.00)
Right rotation						
Accuracy (%)	99.9	(99.9–1.00)	97.2	(96.8–97.5)	>99.9	(99.9–1.00)
AUC	1.00	(1.00–1.00)	1.00	(1.00–1.00)	1.00	(1.00–1.00)

Data are percentages (99% confidence interval) unless otherwise stated. AP=Anterior-Posterior, PA=Posterior-Anterior. AUC=area under the receiver operating characteristic curve.

Appendix Table 9: Results of CXp-Projection-Rotation-Checker based on Histogram of Oriented Gradients feature extraction with a Support Vector Machine classifier

	CheXpert		PadChest		Institution A	
	Internal test		Internal test		External test	
Projection						
Overall Accuracy						
Micro average accuracy (%)	92.3	(91.9–92.8)	89.4	(88.7–90.1)	73.5	(73.1–74.0)
Macro average accuracy (%)	92.3	(91.8–92.8)	89.4	(88.8–90.0)	82.3	(82.1–82.6)
Weighted average accuracy (%)	92.3	(91.9–92.8)	89.4	(88.7–90.0)	80.4	(80.1–80.8)
Frontal						
AP						
Accuracy (%)	93.1	(92.7–93.6)	91.9	(91.4–92.5)	74.9	(74.5–75.3)
AUC	0.99	(0.99–0.99)	0.98	(0.97–0.98)	0.91	(0.90–0.91)
PA						
Accuracy (%)	96.9	(96.6–97.2)	93.9	(93.4–94.4)	81.7	(81.3–82.0)
AUC	0.98	(0.98–0.99)	0.98	(0.98–0.98)	0.93	(0.92–0.93)
Lateral						
Accuracy (%)	94.6	(94.2–95.0)	93.0	(92.5–93.5)	90.4	(90.2–90.7)
AUC	1.00	(1.00–1.00)	0.99	(0.99–1.00)	1.00	(1.00–1.00)
Rotation						
Overall Accuracy						
Micro average accuracy (%)	99.8	(99.7–99.8)	99.2	(99.0–99.4)	>99.9	(99.9–100.0)
Macro average accuracy (%)	99.8	(99.7–99.8)	99.2	(99.0–99.4)	>99.9	(99.9–100.0)
Weighted average accuracy (%)	99.8	(99.7–99.8)	99.2	(99.0–99.4)	>99.9	(99.9–100.0)
Upright						
Accuracy (%)	99.9	(99.8–99.9)	99.6	(99.5–99.7)	>99.9	(99.9–100.0)
AUC	1.00	(1.00–1.00)	1.00	(1.00–1.00)	1.00	(1.00–1.00)
Inverted						
Accuracy (%)	99.9	(99.8–99.9)	99.6	(99.5–99.7)	>99.9	(99.9–100.0)
AUC	1.00	(1.00–1.00)	1.00	(1.00–1.00)	1.00	(1.00–1.00)
Left rotation						
Accuracy (%)	99.9	(99.8–99.9)	99.6	(99.4–99.7)	>99.9	(99.9–1.00)
AUC	1.00	(1.00–1.00)	1.00	(1.00–1.00)	1.00	(1.00–1.00)
Right rotation						
Accuracy (%)	99.9	(99.8–99.9)	99.6	(99.5–99.8)	>99.9	(99.9–1.00)
AUC	1.00	(1.00–1.00)	1.00	(1.00–1.00)	1.00	(1.00–1.00)

Data are percentages (99% confidence interval) unless otherwise stated. AP=Anterior-Posterior, PA=Posterior-Anterior. AUC=area under the receiver operating characteristic curve.

Section S3: References for the Supplementary Appendix

1. Liu Z, Mao H, Wu CY, Feichtenhofer C, Darrell T, Xie S. A convnet for the 2020s. Proceedings of the IEEE/CVF conference on computer vision and pattern recognition, 2022; **2022:** 11976–86.
2. Kingma DP, Ba J. Adam: A method for stochastic optimization. *arXiv preprint arXiv:14126980* 2014.
3. Paszke A, Gross S, Massa F, et al. Pytorch: An imperative style, high-performance deep learning library. *Adv Neural Inf Process Syst* 2019; **32**.
